# Vaccination Status of Mothers and Children from the ‘Mamma & Bambino’ Cohort

**DOI:** 10.3390/vaccines9020168

**Published:** 2021-02-17

**Authors:** Martina Barchitta, Andrea Maugeri, Roberta Magnano San Lio, Maria Clara La Rosa, Claudia La Mastra, Giuliana Favara, Giuliana Giunta, Antonio Cianci, Antonella Agodi

**Affiliations:** 1 Department of Medical and Surgical Sciences and Advanced Technologies “GF Ingrassia”, University of Catania, 95123 Catania, Italy; martina.barchitta@unict.it (M.B.); andrea.maugeri@unict.it (A.M.); roberta.magnanosanlio@phd.unict.it (R.M.S.L.); mariaclara.larosa@unict.it (M.C.L.R.); claudia.lamastra@unict.it (C.L.M.); giuliana.favara@unict.it (G.F.); 2 Obstetrics and Gynecology Unit, Department of General Surgery and Medical Surgical Specialties, University of Catania, 95123 Catania, Italy; giunta.giuliana@studium.unict.it (G.G.); acianci@unict.it (A.C.)

**Keywords:** birth cohort, communicable diseases, pregnancy, vaccine hesitancy, public health

## Abstract

According to the evidence demonstrating vaccines’ safety and effectiveness in anticipation of and during pregnancy, several countries have established immunization programs during the periconceptional period. Here, we evaluated vaccination status among 220 mother–child pairs, using data from the ‘Mamma & Bambino’ cohort. The self-reported data were evaluated at delivery, and with planned follow-ups at 1–2 years after delivery. In general, we noted that the vaccination status among the women was heterogeneous, ranging from 8.3% (vaccine against Human Papillomavirus, HPV) to 65.6% (vaccine against Diphtheria Tetanus and Pertussis, DTaP). Excluding the women who contracted the diseases in the past, the main ground for refusal was the lack of information. We also demonstrated that increasing age was associated with higher odds of not being vaccinated against Measles-Mumps-Rubella (MMR; OR = 1.12; 95% CI = 1.04–1.21; *p* = 0.004), HPV (OR = 1.20; 95% CI = 1.08–1.33; *p* = 0.001) and DTaP (OR =1.09; 95% CI = 1.01–1.18; *p* = 0.040). As expected, we showed that the proportion of newborns vaccinated with the Hexavalent and Pneumococcal vaccines was high (99.5% and 98.6%, respectively), while the vaccination coverage against MMRV did not reach the auspicated threshold (84.1%). Overall, these results underlined the need for the improvement of women’s knowledge about the recommendations for vaccination, especially during pregnancy.

## 1. Introduction

Vaccination is a key strategy for the prevention and control of communicable-diseases [1,2], and therefore vaccines have a crucial role in the public health agenda for improving global health and life expectancy [2]. Indeed, more than twenty life-threatening diseases—such as Tetanus, Pertussis, Diphtheria, Haemophilus influenzae type b, and Meningococcal infections—can now be prevented through accurate vaccine programs [1,3]. For instance, the incidence of several communicable diseases—such as measles and rubella—has declined in recent decades [4]. Similarly, vaccination has not only reduced the incidence of poliomyelitis by 99% [5], it has also led to smallpox’s eradication [6]. More recently, it has been also suggested that more knowledge of evidence-based recommendations could also tackle both emerging infectious diseases and antimicrobial resistance [7,8,9,10]. In this scenario, public health strategies are moving towards a life-course immunization approach, with a particular focus on children and women of childbearing age [11,12,13]. 

As vaccine-preventable diseases (VPDs) can negatively affect fertility and pregnancy outcomes, vaccinations against Measles, Mumps, Rubella, Varicella and Human Papillomavirus (HPV) are indicated for women of childbearing age [14,15]. The high risks arising from maternal infections also make it necessary to vaccinate women against Measles-Mumps-Rubella (MMR) and Varicella in anticipation of pregnancy. Indeed, while the majority of vaccines are safe and effective for pregnant women and the developing fetus [16,17,18,19,20], those containing live attenuated viruses are contraindicated during pregnancy. This is particularly the case for MMR and Varicella vaccines, such that women should already be vaccinated at the onset of pregnancy. Similarly, vaccination against HPV is not currently recommended during pregnancy, but only due to the lack of evidence about its safety in pregnant women. In fact, any accidental administration in this period does not lead to the voluntary interruption of pregnancy. During pregnancy, vaccination against Diphtheria, Tetanus, and Pertussis (DTaP), as well as that against Seasonal Influenza, are proven to be safe, and are hence recommended. According to the Italian National Immunization Plan (Piano Nazionale Prevenzione Vaccinale 2017–2019; PNPV), the recommended period for DTaP vaccination is from the 27th to the 36th week of gestation, while the vaccine against Seasonal Influenza can be administered in any trimester of pregnancy [21]. Despite these recommendations, however, the vaccination coverage among pregnant women remains very low worldwide [20,22,23]. 

Acquired maternal antibodies can be transferred to the fetus, conferring passive immunity until the first infant immunization [24]. The first year of life is crucial to immunize against some of the most important VPDs by administering the first doses of vaccines that—in most cases—require a second booster dose during the second year. As was observed for pregnant women, however, the vaccine coverage among infants and children is not enough to control VPDs in several countries. Accordingly, many of them have been forced to enact mandatory childhood immunization legislation for the prevention of several VPD outbreaks, which instead occurred in the past decade [25,26,27]. In July 2017, for instance, the Italian immunization strategies for infants extended to ten times the number of mandatory vaccines [28,29]. Indeed, newborns are mandatorily vaccinated against Diphtheria, Tetanus, Pertussis, Poliomyelitis, Hemophilus influenzae type b, and Hepatitis B through three doses of the hexavalent vaccine at the 3rd, 5th and 13th months of life [28,29]. In the second year of life—and specifically between the 13th and 15th months—the mandatory vaccination against Measles-Mumps-Rubella-Varicella (MMRV) is carried out by the quadrivalent MMRV vaccine, or by the trivalent MMR vaccine and monovalent Varicella vaccine [28,29]. The other recommended vaccinations are those against Streptococcus pneumoniae (i.e., pneumococcal vaccine), Meningococcal serogroup B and C, Rotavirus, and Seasonal Influenza [28,29]. 

Mandatory vaccination is considered to be a straight-forward solution for addressing the observed insufficient vaccination coverage, an important public health issue that still leads to VPD outbreaks and deaths worldwide [30]. On the other hand, however, there is the worrying phenomenon of vaccine hesitancy, which consists in the “delay in acceptance or refusal of vaccines despite availability of vaccinations services” [1,31]. While vaccine registries alone can provide accurate estimates of vaccination coverage in mothers and their children, researchers have the task of investigating which demographic and social factors may influence vaccination choices. Moreover, both disinformation and a lack of confidence in vaccination play a key role in the determination of the low vaccine coverage among pregnant women [20,31,32,33]. In this field of research, mother–child cohorts represent a resource to investigate the causes and consequences of inadequate vaccination coverage during pregnancy and in the early life period. Here, we used data from the ‘Mamma & Bambino’ cohort to assess the vaccination coverage among pregnant women and their children in Catania, Italy. Furthermore, we evaluated the main determinants involved in vaccination choice, with the intention of informing future strategies against low vaccination coverage and vaccine hesitancy. 

## 2. Materials and Methods

### 2.1. Study Design

We used data from the ‘Mamma & Bambino’ cohort, an ongoing Italian birth cohort, the study design and protocols of which are fully described elsewhere [34,35,36,37,38]. Further information can be also found at the website http://www.birthcohorts.net. The study protocol is in line with the Declaration of Helsinki, and it has been approved by the ethics committees of the involved institutions (the Ethics Committee of the ‘Azienda Ospedaliero-Universitaria Policlinico-Vittorio Emanuele’, and the Ethics Committee ‘Catania 1’; protocol numbers: 47/2014/VE; 48/2015/EMPO; 186/2015/EMPO; 197/2016/EMPO; 213/2017/EMPO; 231/2018/EMPO; 263/2019/EMPO). All of the women are fully informed of the purpose and procedures, and gave written informed consent. The women themselves, or their parents, gave the written informed consent to participate in the study.

From 2014, pregnant women referred to the Azienda Ospedaliera Universitaria Policlinico ‘G. Rodolico-San Marco’ (Catania, Italy) were enrolled during their prenatal obstetric counselling, at the 4th–20th gestational weeks (mean = 16th week). At the recruitment, a structured questionnaire is administered by trained epidemiologists in order to collect information on the participants’ sociodemographic variables and vaccination status. Their educational level is categorized as a low–medium (primary school, i.e., ≤8 years of school) or high education level (high school education or greater, i.e., >8 years of school). The women are also classified as employed or unemployed (including students and housewives). The information on the mothers and their children is also collected through telephone interviews at delivery, and with planned follow-ups at 1 and 2 years after delivery. In the current analysis, we included mother–child pairs with a complete assessment of the maternal sociodemographic characteristics and the vaccination status of the mothers and children until two years of age. 

### 2.2. Data Collection

For the current analysis, the self-reported maternal vaccination status was collected during the follow-up interview at delivery, while the vaccination status of the children was assessed from their mothers during the follow-up interviews at 1 and 2 years. The structured questionnaire aimed to collect information referring to the maternal vaccination status (i.e., vaccinated or non-vaccinated), time of vaccination (i.e., in the past/childhood, in anticipation of pregnancy, or during pregnancy), and grounds for refusal for the following vaccines: (i) MMR, (ii) HPV, (iii) DTaP, (iv) Varicella, and (v) Seasonal Influenza. With respect to the grounds for refusal, a structured multiple choice question was used, including the following options: “having previously contracted the disease”, “lack of confidence in vaccination (i.e., due to fear, anxiety, mistrust etc.)”, and “disinformation (i.e., due to lack of knowledge, lack of counselling by general practitioner or gynecologist, etc.)”. Moreover, we collected information on the childrens’ vaccination status, time of vaccination, and any booster shots for the following vaccines: (i) Hexavalent, (ii) MMRV, and (iii) Pneumococcal. The mothers were asked to provide this information by consulting their vaccination record booklet and that of their children.

### 2.3. Statistical Analyses

Statistical analyses were performed using SPSS software version 26.0 (SPSS, Chicago, IL, USA). Descriptive statistics were used to characterize the mothers using frequency (%), or median and interquartile range (IQR). The characteristics of the children were described using frequency (%), or median and range. Prior to the analysis, the normal distribution of the continuous variables was checked using the Kolmogorov–Smirnov test. Next, the continuous variables underlying the skewed distribution were compared using the Mann–Whitney U test for comparisons between two groups. Logistic regression analysis was used to identify the main determinants of vaccination choice. The results were reported as an Odds ratio (OR), and with a 95% confidence interval (CI). All of the statistical tests were two-sided, and *p*-values of < 0.05 were considered statistically significant.

## 3. Results

### 3.1. Characteristics of Study Population

The present analysis included 220 mother–child pairs (maternal age = 15–45 years, median = 37 years) with a complete assessment of vaccination status, from the ‘Mamma & Bambino’ cohort. All of the women were enrolled at a median gestational age of 16 weeks, from 2014 to 2018. With respect to their social characteristics, more than eight out of ten women reported a high education level (86.4%), while 56.8% were employed.

### 3.2. Vaccination Choice among Women

**Figure 1:**
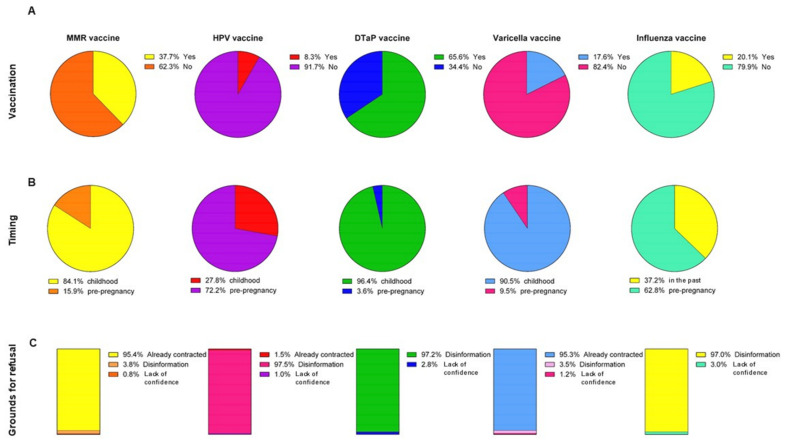
Vaccination status among pregnant women. This panel shows (**A**) the proportion of vaccinated women for MMR, HPV, DTaP, Varicella, and Influenza; (**B**) the proportion of women vaccinated in childhood or during the pre-pregnancy period; and (**C**) the grounds for refusal among the non-vaccinated women.

We first evaluated the vaccination status of the recruited women at delivery. We noted that the percentage of vaccinated women ranged from 8.3% (for the vaccine against HPV) to 65.6% (for the vaccine against DTaP) depending on the type of vaccine (Figure 1A). We next evaluated whether the women were vaccinated in the past/childhood, in anticipation of pregnancy, or during pregnancy. In general, the women indicated their childhood and the period immediately prior to pregnancy as the most common times of vaccination, while none of them planned vaccinations during pregnancy (Figure 1B). For instance, among the women vaccinated for MMR, DTaP and Varicella, the majority were vaccinated in the childhood (84.1%, 96.4%, and 90.5%, respectively). By contrast, a higher proportion of women were vaccinated against HPV and Seasonal Influenza (72.2% and 62.6%, respectively) in anticipation of pregnancy (Figure 1B). We next evaluated grounds for refusal among non-vaccinated women (Figure 1C). Specifically, among those who had not been vaccinated against MMR, 95.4% declared that they already contracted the diseases, while only a small proportion were not informed about the MMR vaccine (3.8%) or were unconfident with it (0.8%). Similarly, 95.4% of the non-vaccinated women against Varicella already contracted the disease, while 3.5% of them were not fully informed about the vaccine and only 1.2% were unconfident with it. By contrast, the main grounds for the refusal of vaccination against HPV, DTaP, and Seasonal Influenza was the lack of information about the vaccines (97.0%, 97.2%, and 97.0%, respectively). 

### 3.3. Vaccination Status of Children

**Figure 2:**
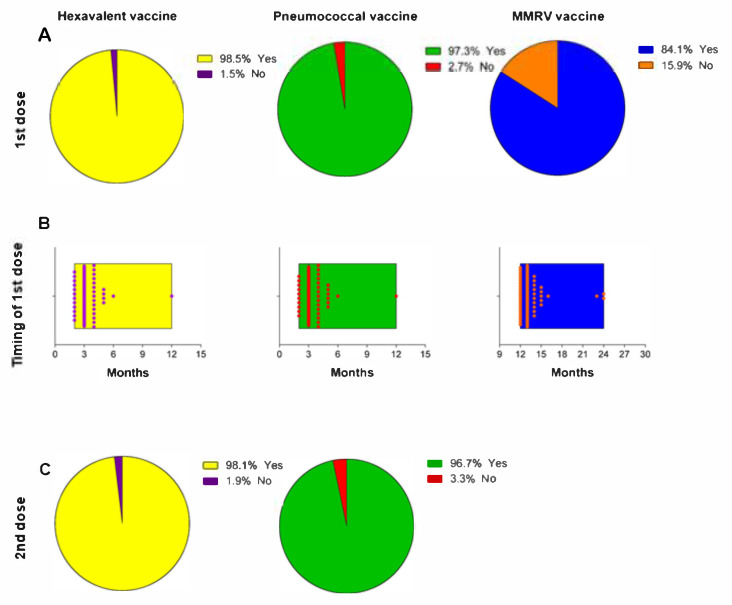
Vaccination status of the children. This panel shows (**A**) the proportion of children who received the first dose of the Hexavalent, Pneumococcal, and MMRV vaccines; (**B**) the timing of the receipt of the first dose; and (**C**) the proportion of children who received the second dose.

We next assessed the vaccination status among the children, using the information received from their mothers during the follow-up interviews. Figure 2A shows that the percentage of vaccinated children was 98.5% for Hexavalent, 97.3% for Pneumococcal and 84.1% for MMRV. Specifically, many children received the first dose of the vaccine according to the recommended period for the Hexavalent and Pneumococcal vaccines (a median of 3.0 months; IQR = 2.0–12.0), as well as for the MMRV vaccine (a median of 13.0 month (IQR = 12.0–24.0) (Figure 2B). We also observed a high proportion of children who received the booster shots for the Hexavalent (98.1%) and Pneumococcal (96.7%) vaccines (Figure 2C).

### 3.4. Association between Age and Vaccination Choice

**Figure 3:**
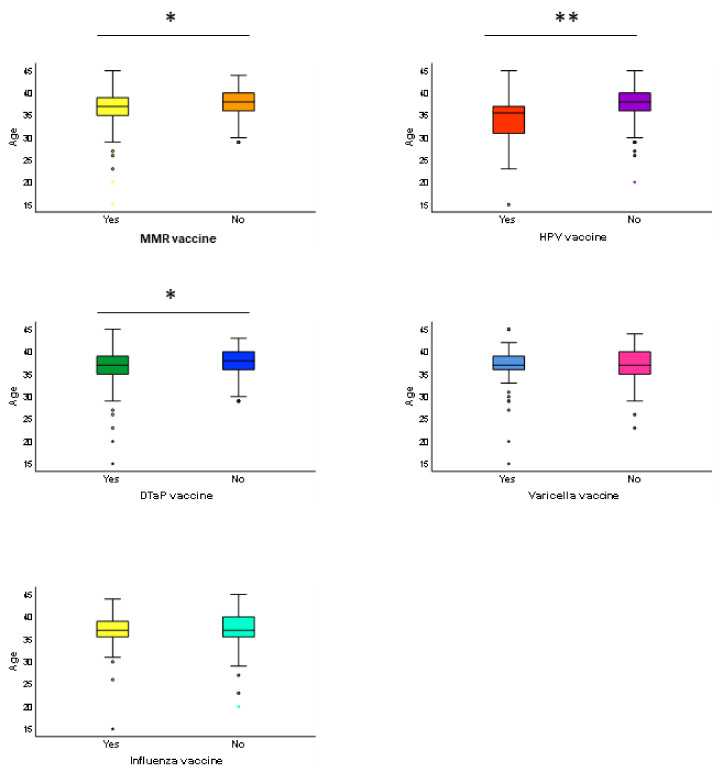
Comparison of age between the vaccinated and non-vaccinated women. These box plots show the distribution of age between the vaccinated and non-vaccinated women. Their age was compared using the Mann–Whitney U test. * *p*-value < 0.05; ** *p*-value < 0.01.

Next, we aimed to test the association of age, educational level and employment status with vaccination choice among the mothers. Accordingly, we noted that the women who were unvaccinated against MMR, HPV and DTaP were older compared with their vaccinated counterparts (Figure 3). Notably, the logistic regression model further demonstrated that increasing age was associated with higher odds of not being vaccinated for MMR (OR = 1.12; 95% CI = 1.04–1.21; *p* = 0.003), HPV (OR = 1.18; 95% CI = 1.07–1.30; *p* = 0.001) and DTaP (OR = 1.09; 95% CI = 1.01–1.81; *p* = 0.044). Moreover, the positive association between age and non-vaccination for MMR (OR = 1.12; 95% CI = 1.04–1.21; *p* = 0.004), HPV (OR = 1.20; 95% CI = 1.08–1.33; *p* = 0.001) and DTaP (OR = 1.09; 95% CI = 1.01–1.18; *p* = 0.040) remained significant after adjusting for educational level and employment status. However, no association between social factors and vaccination choice was evident.

## 4. Discussion

In Europe, the recommendations and decision-making for vaccination policies are different across the different countries [39,40]. In order to level out these differences and to prevent millions of deaths, the Global Vaccine Action Plan 2011–2020 has suggested that all countries should have reached ≥ 90% of their national coverage for all mandatory and recommended vaccines by 2020 [3]. Nevertheless, in the same period, several European countries have registered a decreasing trend in their vaccination coverages, with alarming outbreaks of VPDs [41,42]. Although the global immunization strategy involves people of all ages, pregnant women and their children are considered to be two major groups on which to focus efforts and interventions from public health professionals [14,15]. 

In the current study, we first assessed the vaccination status of mothers and children enrolled in ‘Mamma & Bambino’ cohort. Despite the existing recommendations from the Italian Ministry of Health [21,43], the vaccination coverage among pregnant women did not reach the auspicated threshold, as only 20%–65% of them were vaccinated against DTaP, MMR, Seasonal Influenza, and Varicella. Notably, the coverage was modest for the DTaP vaccine (approximately 65%) and low or very low for MMR (~38%), Seasonal Influenza (~20%), and Varicella (~19%). However, the vaccines against the above-mentioned diseases are considered safe and useful for promoting health among women of childbearing age and in anticipation of pregnancy [21,43]. The vaccination against HPV deserves separate discussion, as the Italian PNPV introduced, in 2007, the recommendation for vaccinating against HPV to all the adolescents aged 12 to 13 years. However, this recommendation had no or little impact on our findings, due to the age distribution of our study population. Indeed, the majority of the recruited women were aged 20 years or older, and hence they were not the target of this vaccination strategy. Accordingly, only about 8% of pregnant women were vaccinated against HPV, and most of them were vaccinated in anticipation of pregnancy. 

As discussed above, it is now well known that contracting several VPDs during pregnancy is associated with the increased risk of complications both in mothers and newborns, including adverse pregnancy outcomes, longer hospitalization periods, and higher mortality rates [20]. In spite of this, however, we observed that no woman planned to be vaccinated during pregnancy. This is particularly important for vaccines that are not contraindicated in pregnancy, such as those against DTaP and Seasonal Influenza. Overall, our findings are in line with the low vaccination coverage reported also in other countries [22,23]. A plausible explanation lies in the failure of vaccination programs at the national and international levels. Indeed, the success of these programs depends on a lot of social and organizational factors, which might be associated with knowledge on and confidence in vaccination at the childbearing age and during pregnancy. For this reason, previous studies have been conducted in order to determine the awareness of vaccines’ usefulness among pregnant women, considering their key role against VPDs [44,45,46,47,48]. In this context, so-called ‘vaccine hesitancy’ is a widespread behavior influenced by: confidence, as a low level of trust in the vaccine; complacency, as a low awareness of the vaccine’s usefulness and safety; and convenience, as a lack of an accessible healthcare system [32,49]. In line with this evidence, we next investigated the main grounds for the refusal of vaccination among pregnant women. Beyond those who already contracted the diseases, the main grounds for refusal was the lack of information about vaccines and vaccination programs, while only small proportions declared themselves to be unconfident in them. Notably, no woman reported that they were to be informed about the existing recommendations in anticipation of and during pregnancy. This suggests the need for increasing knowledge and counselling in women who intend to get pregnant. These findings—though negative—are partially encouraging, because they leave room for improvements of activities raising awareness and medical counselling. Instead, it would have been more challenging to deal with fear, anxiety, and mistrust. 

Beyond the reasons behind the choice of not getting vaccinated, it would be important to identify those social factors underpinning the lack of knowledge and confidence with vaccination [50]. Indeed, the social determinants vary among countries of different income levels, supporting the idea that their effects on vaccination choice should be considered in order to design appropriate interventions [33]. In order to evaluate which social factors affect vaccination choice, we tested the association of age, educational level and employment status with vaccination status among pregnant women. Interestingly, we noted that the proportion of non-vaccinated women increased with increasing age for the MMR, HPV and DTaP vaccines. This result remained significant after adjusting for educational level and employment status, which were not per se associated with vaccination status. A plausible explanation is that the younger women have probably benefited more from vaccination programs and campaigns than the older ones. However, we hypothesize that age is not a direct determinant of vaccination choice, but rather that this association reflects an improvement in the national vaccination program over the years. On the other hand, these results raise the need for giving more support and attention to women who plan to become pregnant at an older age. It is also worth mentioning the absence of association between age and vaccination against Varicella and Seasonal influenza. Despite their similarity, in fact, it is not clear why age was associated with MMR vaccination but not with Varicella vaccination. In our opinion, the different ratios between vaccinated and non-vaccinated women could reduce the statistical power of this test, and hence we cannot completely rule out a potential relationship. Thus, further studies should be encouraged in order to solve this question. With respect to vaccination against Seasonal Influenza, instead, we recognize that it is completely different from the previous examples in terms of the vaccination schedule in general, and in the awareness of its benefits for pregnant women in particular. Moreover, the vaccination coverage might be, for the most part, influenced by the seasonality of pregnancies. 

Finally, we assessed the vaccination status among children, using the information received from their mothers during the follow-up interviews. As expected, almost all of the children were vaccinated with the Hexavalent (~99%) and Pneumococcal (~98%) vaccines, and according to the times scheduled by the PNPV. However, the vaccination coverage against MMRV (~84%) did not reach the auspicated threshold of at least 90%, despite the re-emerging measles outbreaks across Europe [28,51,52]. It seems unreasonable, but, in 2017, nearly 80% of the children who contracted measles had not been vaccinated [53]. As reported by Jean-Claude Juncker, President of the European Commission, “It is unacceptable that in 2017 there are still children dying of diseases that should long have been eradicated in Europe” [53]. In order to address this issue, in 2017, Italy introduced a new law (n.119/2017) to require ten mandatory vaccines for access to kindergarten, and to primary and secondary schools [29]. Although all of the Italian regions are currently far from the auspicated thresholds, this law has determined an increase in vaccination coverage for both the mandatory (e.g., Hexavalent and MMR) and recommended (e.g., Pneumococcal and Meningococcal C) vaccines [53], maintaining the same trend in 2018 [54]. In line with this, our results indicated that nearly 100% of the children received the booster shots for the Hexavalent and Pneumococcal vaccines, while nearly 100% of them had not yet received those for MMRV, as recommended by PNPV [21]. In spite of these findings, however, further efforts are needed in order to maintain conscious and proactive adherence to the immunization programs, worldwide. For instance, in 2019, nearly 6 million were partially vaccinated for DTaP, without completing the required three dose schedule in the first year of life [55]. 

Our study had some limitations that should be considered. Firstly, the data on vaccination status were self-reported through face-to-face or telephone interview, which did not preclude potential reporting errors, and may suffer from inaccuracies. Although the mothers were asked to report vaccination status by consulting the vaccination record booklet, we cannot compare their response to the national vaccination registry. Secondly, the low sample size did not allow us to adjust for potential confounders, or to rule out the possibility of bias from residual unknown or unmeasured factors. 

## 5. Conclusions

In conclusion, we noted modest or low vaccination coverages among pregnant women, which mainly depended on the lack of information about vaccines and vaccination programs. For this reason, it is necessary to increase knowledge about vaccination among women who plan to become pregnant, and through an improvement of medical counselling. The same applies for the vaccination of newborns, especially against MMRV. To do that, demographic and social determinants should be assessed and considered, in order to understand the concerns and barriers that might affect correct vaccination choice.

## Data Availability

The data presented in this study are available on request from the corresponding author.
